# Deep learning algorithm-enabled sediment characterization techniques to determination of water saturation for tight gas carbonate reservoirs in Bohai Bay Basin, China

**DOI:** 10.1038/s41598-024-63168-8

**Published:** 2024-05-28

**Authors:** Xiao Hu, Qingchun Meng, Fajun Guo, Jun Xie, Eerdun Hasi, Hongmei Wang, Yuzhi Zhao, Li Wang, Ping Li, Lin Zhu, Qiongyao Pu, Xuguang Feng

**Affiliations:** 1 School of Natural Resources, Faculty of Geographical Science, Beijing Normal University, Beijing, 100875 China; 2 Exploration and Development Research Institute, PetroChina Huabei Oilfield Company, Renqiu, 062552 China; 3 College of Earth Science and Engineering, Shandong University of Science and Technology, Qingdao, 266590 China; 4 China Petroleum Engineering & Construction Corp. North China Company, Renqiu, 062552 China; 5 College of Computer Science and Engineering, Shandong University of Science and Technology, Qingdao, 266590 China

**Keywords:** Water saturation, Tight gas carbonate reservoirs, Sediment characterization, Deep learning, Petrophysics, Machine learning, Solid Earth sciences, Geology, Energy science and technology, Fossil fuels, Crude oil

## Abstract

Understanding water saturation levels in tight gas carbonate reservoirs is vital for optimizing hydrocarbon production and mitigating challenges such as reduced permeability due to water saturation (Sw) and pore throat blockages, given its critical role in managing capillary pressure in water drive mechanisms reservoirs. Traditional sediment characterization methods such as core analysis, are often costly, invasive, and lack comprehensive spatial information. In recent years, several classical machine learning models have been developed to address these shortcomings. Traditional machine learning methods utilized in reservoir characterization encounter various challenges, including the ability to capture intricate relationships, potential overfitting, and handling extensive, multi-dimensional datasets. Moreover, these methods often face difficulties in dealing with temporal dependencies and subtle patterns within geological formations, particularly evident in heterogeneous carbonate reservoirs. Consequently, despite technological advancements, enhancing the reliability, interpretability, and applicability of predictive models remains imperative for effectively characterizing tight gas carbonate reservoirs. This study employs a novel data-driven strategy to prediction of water saturation in tight gas reservoir powered by three recurrent neural network type deep/shallow learning algorithms—Gated Recurrent Unit (GRU), Recurrent Neural Networks (RNN), Long Short-Term Memory (LSTM), Support Vector Machine (SVM), K-nearest neighbor (KNN) and Decision tree (DT)—customized to accurately forecast sequential sedimentary structure data. These models, optimized using Adam's optimizer algorithm, demonstrated impressive performance in predicting water saturation levels using conventional petrophysical data. Particularly, the GRU model stood out, achieving remarkable accuracy (an R-squared value of 0.9973) with minimal errors (RMSE of 0.0198) compared to LSTM, RNN, SVM, KNN and, DT algorithms, thus showcasing its proficiency in processing extensive datasets and effectively identifying patterns. By achieving unprecedented accuracy levels, this study not only enhances the understanding of sediment properties and fluid saturation dynamics but also offers practical implications for reservoir management and hydrocarbon exploration in complex geological settings. These insights pave the way for more reliable and efficient decision-making processes, thereby advancing the forefront of reservoir engineering and petroleum geoscience.

## Introduction

### Inherent challenges for hydrocarbon production in tight gas reservoirs

Tight gas reservoirs pertain to a category of natural gas resources that are confined within a rock structure characterized by a relatively low capacity for fluid conductivity due to compact pore-fluid networks. The production of natural gas from these reservoirs is challenging because of the barriers that hinder the flow of gas to the wellbore^1^. Despite the challenges, tight gas reservoirs have vast reserves of natural gas that are economically viable if the production challenges can be overcome. The limited level of porosity and permeability present in tight gas reservoirs poses a significant challenge for the efficient flow of gas through them. To put it differently, the intricate geological structure of these reservoirs restricts the flow of gas within them, making it arduous to extract the valuable resource^2^. The factors that impact gas relative permeability in these reservoirs involve a complex interplay of factors that affect fluid flow in porous rock matrix, with a focus on water saturation (SW) and water-wet carbonate rock. To understand these roles, it is important to first understand the concepts of porosity and permeability. Porosity refers to the amount of space within a rock or sediment that is filled with voids. Tight gas reservoirs inherently have many limitations in porosity and pore trough passages for smoothing the flow^3^.

Permeability, on the other hand, refers to the ease with which fluids can move through those voids. When multiple fluids are present within a constrained passage, the range of movement for a specific fluid is restricted, particularly in instances where there are porous media in tight gas reservoirs^4^. This is where the principle of relative permeability of fluid in relation to its counterparts assumes a critical role in this context^5^. The concept of relative permeability pertains to the proportion of fluid that can pass through a porous medium in relation to the total porosity of said medium. This implies that only a portion of the available pore space can be utilized for fluid flow^6,7^.

In a tight gas reservoir, SW plays an important role in determining gas relative permeability. In such reservoir where there are several fluids present like oil, gas, and water, SW is known as the fraction of pore space occupied by water while total porosity is the volume of pore space present in the system^8^. When water is present in the reservoir, it can block the flow of gas through the rock due to its higher viscosity and lower mobility than gas, which means that it is more difficult for it to move through the pores and fractures in the rock. As a result, gas relative permeability decreases as SW increases^9^.

Reservoir rock wettability is another aspect that aggravates the complexity of flow behavior in the porous environment of tight gas reservoirs. The wettability of the internal surfaces of rock pores affects the relative permeability of the fluids^10^. Water-wet condition refers to the tendency of water to wet the rock surfaces of the reservoir instead of oil and gas. In rocks with inherent water-wet properties, the effective permeability of water increases while that of oil and gas decreases, making it harder for the oil and gas to flow through the rock^11^. This effect is because the water phase would have higher mobility than the other two phases. In contrast, in rocks with inherent oil-wet properties or gas-wet, the oil and gas phase would have higher mobility than the water phase. In this case, the effective permeability of the oil and gas phases would increase while that of water decreases, leading to faster oil and gas production rates^12^.

### Tight gas carbonate reservoir: fluid transition complexity

Carbonate rocks are typically made up of minerals such as calcite and dolomite, which have a natural affinity for water. This means that when the rock is exposed to a mixture of gas and water, the water will tend to adhere to the surface of the rock while the gas flows around it^13^. As a result, the permeability of the rock to gas decreases, because the gas is being forced to flow through a smaller area. On the other hand, on average, carbonate reservoirs have lower porosity and permeability than sandstone reservoirs. Understanding these factors is key to optimizing oil and gas production in reservoirs^14^. The complexity of governing roles for tight gas reservoir fluid flow in porous matrix is a multifaceted process that encompasses various factors, including SW and the water-wet nature of carbonate rock, which impact gas relative permeability^15^. By comprehending how SW and rock wettability affect gas relative permeability, engineers can develop effective techniques for enhancing gas production from tight gas reservoirs.

### Modeling reservoirs: understanding water saturation

Water saturation calculations are essential in petroleum engineering because they form the basis for many engineering concepts. For example, engineers use SW calculations to determine the porosity of a reservoir, which is a measure of how much empty space exists in the rock that makes up the reservoir^16^. Porosity is an important factor in determining how much oil can be extracted from a reservoir and what type of extraction method will be most effective. In addition to porosity, SW calculations are also used to determine other important parameters such as permeability, which is a measure of how easily fluids can flow through the rock^17,18^. This information is essential for designing production wells and determining the best locations for them. Therefore, SW calculations are an essential part of petroleum engineering models. They provide engineers with critical information about the properties of a reservoir and help them to make informed decisions about how to extract oil from it. By accurately calculating SW, engineers can optimize production and increase recovery rates, ultimately leading to more efficient and cost-effective operations.

### Measuring water saturation: techniques and limitations

For many years, accurately predicting SW in subterranean sedimentary layers through petrophysical logging data has been a critical task in the oil and gas industry. Precise estimation of SW is imperative for effective reservoir characterization, productivity analysis, and informed management decisions concerning the reservoir^19^. The traditional methods of predicting SW levels have been time-consuming, cumbersome, and often prone to errors. However, the utilization of machine learning techniques in forecasting of SW has emerged as a promising strategy, making the process much more efficient and accurate^20^. Petroleum engineers usually use petrophysical logging data to determine the properties of reservoir rocks. A petrophysical log records the readings of various tools that measure characteristics such as resistivity, porosity, and density of rock formation^21^. Using these measurements of subsurface formations, geoscientists need to estimate various petrophysical parameters, including SW to complete the reservoir modeling process accurately^22^. The traditional SW calculation methods based on core data or well tests were considered reliable but only valid for specific wells. These methods have limitations, such as sampling bias, data uncertainty, and data sparsity. Additionally, calculations take several weeks or even months, which delays decision-making processes. These challenges led petroleum engineers to explore machine learning and artificial intelligence (AI) approaches to improve the accuracy and speed of SW predictions.

### Smart predictions: machine learning for water saturation

Machine learning has proven to be a viable tool for SW prediction^23–25^. Essentially, machine learning algorithms take input data, learn from it, and make a prediction or output. In the case of petrophysical logging, the input data comprises various petrophysical log attributes and core data provided in supervised learning technique. The output is a prediction of SW. With a machine learning algorithm, the relationship between the input data and output is modeled, which can greatly improve accuracy. Machine learning algorithms work on a vast amount of data to develop a relationship between petrophysical log parameters and SW levels^24^. This relationship modeling makes these models more accurate, and they create a prediction that can capture the condition of the reservoir structure more efficiently than traditional methods. It also makes use of past data to make predictions that are easily adaptive to changes in the dynamics of the rock formations. Machine learning algorithms have the potential to identify patterns and relationships in large and complex datasets^26^. The use of artificial neural networks and deep learning (DL) architectures can learn multiple features that enhance the prediction of SW levels under different geological environments. Additionally, unsupervised learning techniques can be useful in identifying patterns and clusters from vast quantities of unsupervised data. In conclusion, the application of machine learning models in predicting SW in sediment underground layers through petrophysical logging data offers a more efficient and effective approach to solving current challenges^27^.

The aim of the article is to explore the pivotal role of water saturation (SW) in the production of tight gas reservoirs, focusing specifically on the complex interactions involved in effective drive mechanisms of production fluid in such reservoirs. This research utilizes a substantial dataset comprising a total of 33,950 data points from the Shulu Sag of the Bohai Bay Basin in China, to improve computational accuracy. The key parameter utilized in this study is SW, with inputs including Density index (RHOB), Corrected Gamma Ray (CGR), Sonic Transition Time (DT), Neutron porosity index (NPHI), Potassium content (POTA), Uranium content (URAN), Thorium content (THOR), and photoelectric coefficient index (PEF).

In contrast to traditional machine learning models that use statistical approximation techniques and gradient optimization methods, this study leverages structured and adapted deep/shallow learning algorithms such as Gated Recurrent Unit (GRU), Recurrent Neural Networks (RNN), Long Short-Term Memory (LSTM), Support Vector Machine (SVM), K-nearest neighbor (KNN) and Decision tree (DT), which are specifically designed to handle sequential and temporal patterns, making them ideal for analyzing time series geological data and repetitive time-dependent sediment sequences. Deep learning models have shown superior performance in capturing complex patterns and dependencies in sequential data compared to classical machine learning approaches. GRU, RNN, LSTM, SVM, KNN and DT are particularly well-suited for handling time series data due to their ability to retain long-term dependencies and model temporal relationships effectively. By incorporating these state-of-the-art deep learning algorithms, this study aims to enhance the accuracy and efficiency of detecting sequential and temporal patterns in geological data analysis. This approach allows for more precise identification of trends and patterns in time-dependent sediment sequences, leading to better insights and decision-making in the field of geology and earth sciences.

RNNs algorithms are powerful deep learning algorithms for sequential data processing, yet they have certain limitations. RNNs suffer from vanishing and exploding gradient problems, hindering the learning of long-range dependencies. LSTMs and GRUs address these issues to some extent but are still prone to overfitting on small datasets and struggle with capturing very long-term dependencies. To control these limitations, techniques such as regularization methods like dropout, batch normalization, and weight decay can help alleviate overfitting.

**Figure 1:**
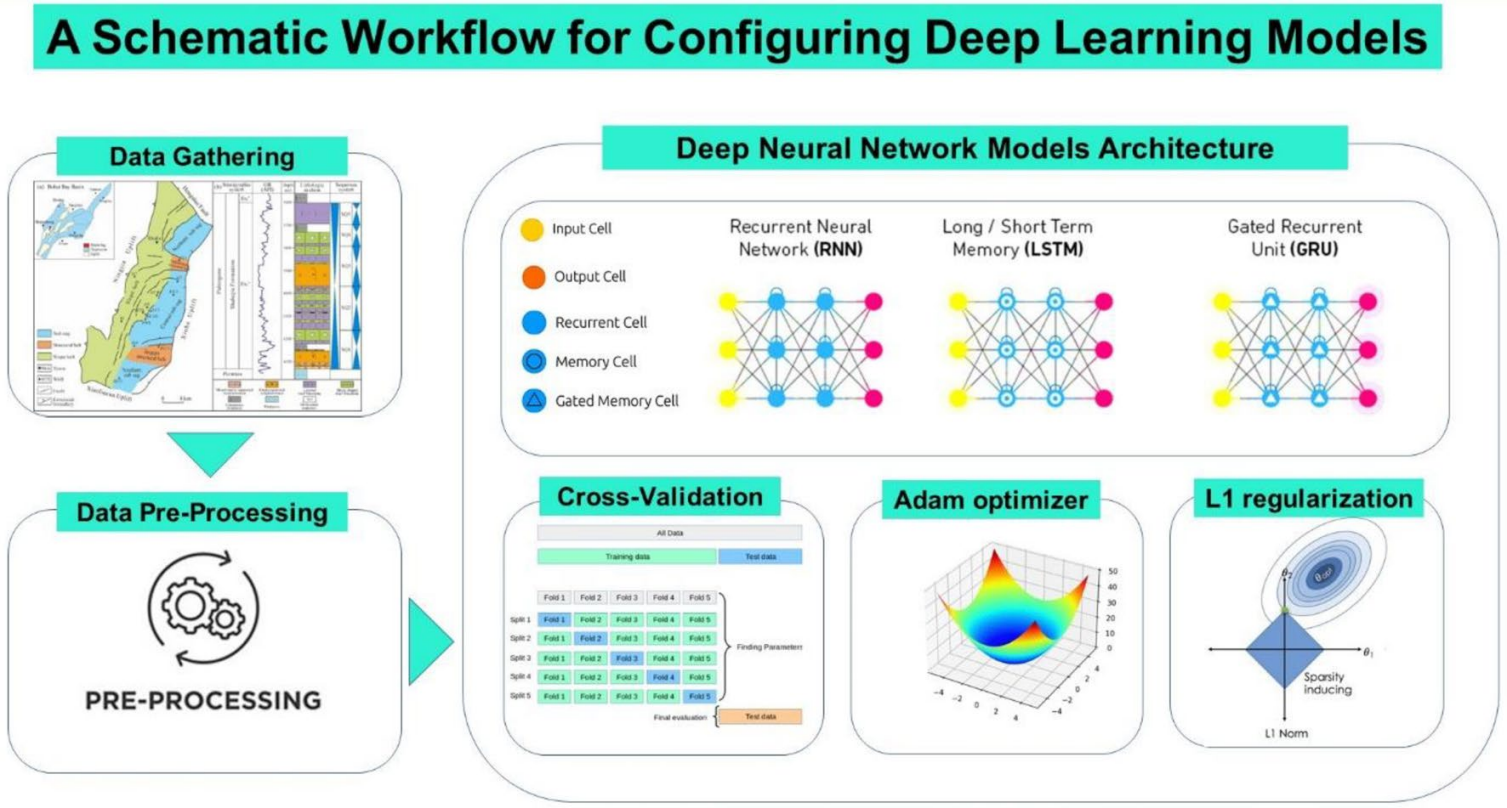
A workflow for configuring deep learning models.

Figure 1 presents the workflow for configuring deep learning models.

## Research background

Oil exploration and production companies require accurate and precise predictions of hydrocarbon reservoir behavior to optimize their production. Among the most valuable tools for making such predictions are well logging techniques, which enable measurement of different petrophysical properties in reservoir rocks. One such property is SW, which is essential for determining the extent of hydrocarbon reserves in a reservoir. However, predicting SW accurately is a challenging task due to the complexities of tight gas reservoirs. Laboratory techniques based on core evaluation are still highly reliable in research related to determining the sedimentary characteristics of unconventional tight gas reservoir zones. For example, Al-Mudhafar used probabilistic neural networks (PNNs) for lithofacies classification and generalized boosted regression model (GBM) for core permeability modeling, achieving high accuracy and overcoming multicollinearity^28^. Al-Mudhafar utilized Bayesian model averaging (BMA) and least absolute shrinkage and selection operator regression (LASSO) for core permeability modeling, achieving accurate predictions comparable to conventional regression analysis^29^. Anifowose et al. explored ML techniques for permeability estimation in a Middle Eastern carbonate reservoir, integrating seismic attributes and wireline data. Depth-matched datasets yielded marginally improved predictions, aiding informed ML technique selection for reservoir characterization^30^. Al-Mudhafar applied probabilistic neural networks for lithofacies classification and smooth generalized additive models (SGAM) for permeability modeling, enhancing accuracy and preserving reservoir heterogeneity in South Rumaila oil field^31^. Radwan et al. examine the lithological characteristics, SW, porosity network, and petrophysical characteristics using core samples, thin sections, well logging data, and laboratory measurements. The findings indicate that the examined geological formation exhibits characteristics of both unconventional and conventional reservoirs, influenced by a combination of depositional and diagenetic mechanisms that affect its petrophysical properties. Moreover, the investigation establishes a correlation between petrophysical parameters and sediment microfacies, and highlights the disparity between source and reservoir rocks^32^. Kamali et al. used machine learning models—group method of data handling (GMDH), polynomial regression (PR), support vector machine (SVM), and decision tree (DT)—to predict permeability in heterogeneous carbonate gas condensate reservoirs, achieving improved accuracy over empirical correlations^7^. Makarian et al. highlight the use of rock physics feature templates and models to characterize hydrocarbon reservoirs more accurately and quickly. The authors' primary focus is on a carbonate tight reservoir located in the southwestern region of Iran. They utilize well-logging data to thoroughly analyze the lithology and pore fluid saturation of the reservoir. Results show that RPT is an effective approach to organizing data and analyzing fluid distribution, with the high oil saturation causing a decrease in seismic velocities and impedances^33^. Zhou et al. investigate SW in tight sandstone reservoirs, which can be challenging due to their complex pore structures caused by strong diagenesis. The authors use various analytical techniques to classify diagenetic facies, study conduction mechanics, and develop a new saturation model based on these factors. Deep learning Sophisticated neural networks are utilized to improve the precision of the model structure. The proposed model is validated using core samples from a Permian block in the eastern margin of the Ordos Basin and shows significant improvement in saturation evaluation accuracy^34^. The research Zou et al. focus on the geological characteristics and criteria for identifying tight gas sandstone reservoirs in various basins in China, including their genetic origins, lithology, diagenetic evolution, porosity, permeability, and heterogeneity. The study synthesizes data from known tight gas accumulations in the Ordos, Sichuan, Tuha, and Songliao basins to establish criteria for recognizing these reservoirs in China^35^.

To address this challenge, many researchers have looked into applying machine learning (ML) and DL algorithms to petrophysical well logging data to predict SW in tight gas reservoirs. Several research studies have explored the application of ML and DL on predicting SW in tight gas reservoirs. Feng et al. focus on accurately simple machine learning calculating SW in tight sandstone reservoirs, specifically within the Penglaizhen Formation situated in the Shifang gas field. The traditional methods of Archie formula, porosity log, and Density log have proven to have large errors due to complex pore structures and high-water saturation. Therefore, the authors proposed a refined Gaussian process regression (GPR) technique to improve SW calculation accuracy for modeling^36^. Baziar et al. used support vector machine (SVM), multilayer perceptron (MLP), decision tree (DT), random forest (RF), and Extreme Gradient Boosting regression (XGBoost) methods to predict SW in Mesaverde tight gas sandstones. Support Vector Machine models generally outperformed others, although exceptions existed, with correlation coefficients ranging from 0.6 to 0.8^37^. Movahhed et al. explored techniques for Sw estimation in carbonate reservoirs, introducing a method for cementation factor calculation in Archie formula. multi-dimensional dot-pattern recognition (MRGC), fuzzy logic, and novel crossplot techniques were used, yielding variable cementation factors correlated with effective porosity^38^. A deep-learning model was created by Zhang et al. to tackle intricate subsurface 2D oil/water two-phase flow partial differential equations (PDEs) in reservoir engineering utilizing a sophisticated PDE solution architecture, Fourier neural operator (FNO). The researchers contemplated a variety of factors such as SW and porosity, handpicked important variables, augmented dimension channel, expanded the network's structure, and successfully solved the engineering issue^39^. Otchere et al. used enhanced data analytics and XGBoost to accurately predict petrophysical parameters of reservoirs from wireline logs obtained from the Volve field in the North Sea, and proposed a new ensemble model that performed better than traditional models, demonstrating the potential for ensemble modeling to enhance reservoir characterization^40^. Rashid and colleagues have conducted a meticulous investigation into the petrophysical properties of carbonate sediments containing gas reserves at the Qadirpur field, located in the Central Indus Basin of Pakistan. The study used sophisticated machine learning techniques, specifically self-organizing map (SOM) and cluster analysis, to provide a comprehensive assessment. The study found that reservoir quality varied throughout the Eocene rock units, with some reservoirs having high effective porosity and hydrocarbon saturation^41^. Ibrahim et al. applied artificial intelligence (AI) techniques, utilizing artificial neural networks (ANNs) and adaptive neuro-fuzzy inference system (ANFIS), to predicted Sw from conventional well logs. Both models achieved accurate Sw estimation with R values exceeding 0.90 and AAPE less than 5%. The study introduced a new empirical correlation derived from ANN biases and weights, validated with unseen data, providing a reliable method for Sw prediction without costly core analyses^42^. Markovic and colleagues (2022) conducted a thorough examination using the XGBoost technique to ascertain inter-pore SW within Canadian sandstone oil resources through the analysis of LF-NMR and density information^43^. Miah et al. focus on the using of machine learning tools in estimating reservoir properties for hydrocarbon production through log-based reservoir characterization. The study aims to recognize and categorize influential log variables to estimate SW through the MLP and least-squares support vector machine (LS-SVM) algorithms. The study findings indicate the order of importance of influential logging variables and provide a strategy to reduce exploration costs through SW forecasting using fewer log variables^44^. Feng et al. investigated Sw estimation in Penglaizhen Formation tight gas reservoirs, identifying challenges due to complex pore structure. They proposed an optimized GPR model using AC, EDN, XI, and φe parameters. The GPR model exhibited superior accuracy, especially in complex reservoirs, promising broad applicability^36^. Wood introduces a new and innovative approach to data matching that utilizes transparent open box machine-learning techniques for the purpose of predicting petrophysical metrics of oil and gas reservoir sections. This approach involves the integration of standardized well log curves with lithofacies and stratigraphic information, resulting in a more comprehensive and accurate analysis. The proposed method demonstrates high prediction accuracy and detailed prediction error analysis, making it superior to existing regression models or correlation-based optimized machine learning models^45^.

Despite the current research emphasis on the application of ML solutions in predicting SW, few studies have evaluated the effectiveness of these techniques in tight gas reservoirs using real-world petrophysical well logging data. Therefore, this study aims to fill this gap by applying different ML techniques on the petrophysical well logging data from the tight gas reservoir of lacustrine carbonate rocks in the lower sub-member of the third member of the Shahejie Formation (Es_3_^x^) in the Shulu Sag of the Bohai Bay Basin, China. By comparing the prediction results with actual values, the most accurate and reliable model will be identified.

## Methodology

### Geological setting

**Figure 2:**
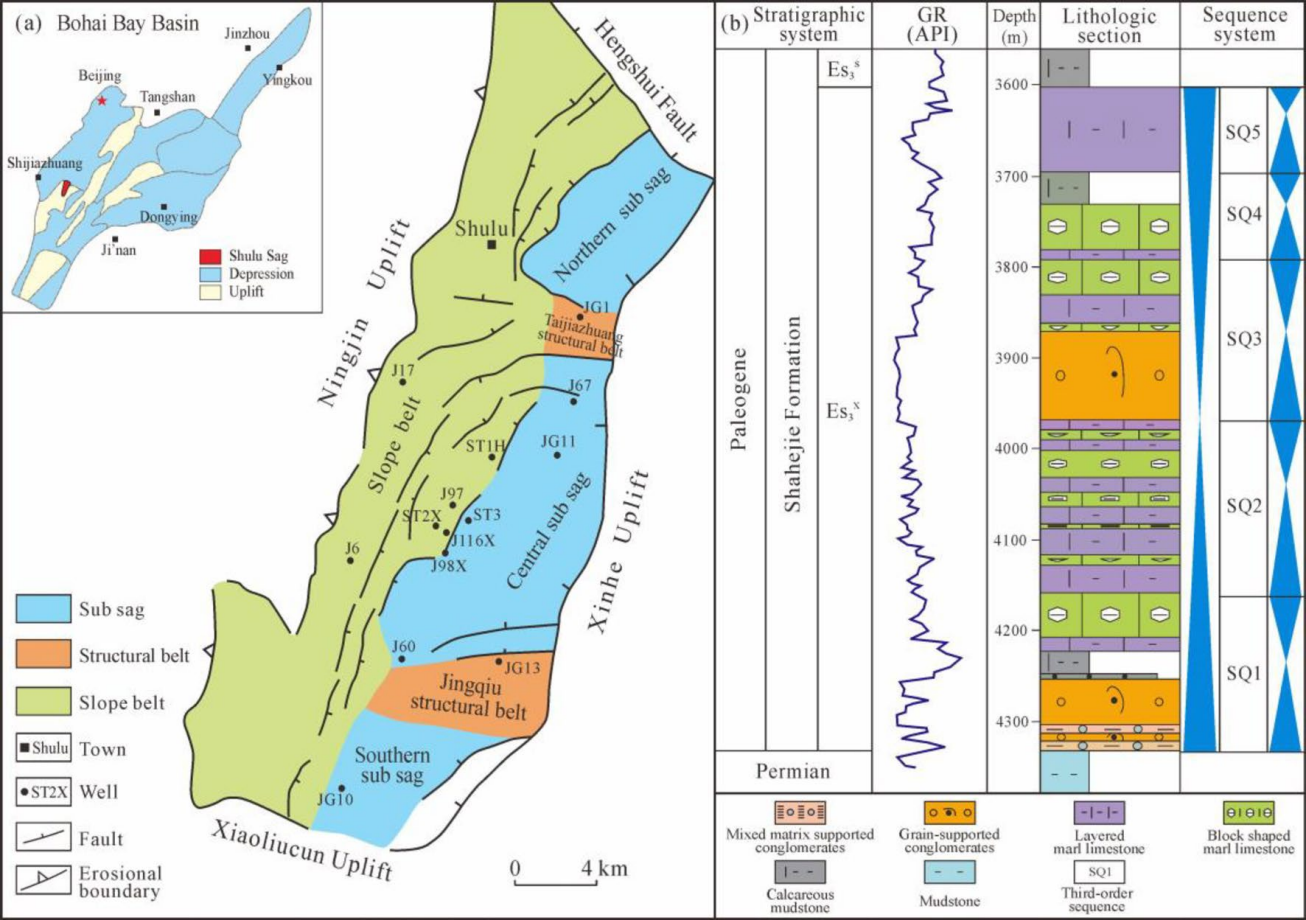
Comprehensive geological map of Shulu Sag. (**a**) Location and structural units; (**b**) Stratigraphic system and lithologic section map. Using CorelDRAW 2018 by the authors (https://www.corel.com/).

The Shulu Sag, situated in the Jizhong Depression within the Bohai Bay Basin, is a fault half-graben depression with a northeast trend. It comprises six structural unit belts, featuring various formations from the Neogene and Paleogene periods. During the sedimentation phase of the Shahejie Formation's third member (Es_3_), the basin was divided into three sub-sags controlled by ancient uplifts. The focus of this study is on the lacustrine marl limestone reservoir within the Es_3_^x^ sub-member, characterized by its deep lake sediments and complex lithology including conglomerates and marl limestone. The reservoir's poor performance is attributed to its low porosity and permeability, with porosity levels ranging from 0.5 to 2.5% for marl limestone and 1.0 to 5.0% for conglomerates, and permeability generally below 10 × 10^−3^ μm^2^. Previous research indicates that marl limestone and terrigenous conglomerates are significant lithologies for tight oil and gas reservoirs, leading this study to focus solely on petrophysical assessments of the marl limestone reservoir. Figure 2 presents the geographical location of the studied field, the stratigraphic system, and the sequence column of geological formations.

In this article, three deep learning algorithms—GRU, RNN, and LSTM—are utilized to predict SW. The review's emphasis on the necessity of employing deep learning algorithms instead of shallow ones is crucial in assessing the validity of our approach. Consequently, we incorporated three shallow models of classical learning algorithms to juxtapose the performance and accuracy of the modified version, thereby justifying the selection of deep learning algorithms in our study.

While shallow learners, such as traditional regression models or decision trees, can handle simpler patterns in the data, the geological complexities and nonlinear relationships inherent in tight gas carbonate reservoirs demand a more sophisticated approach.

- The intricate data patterns within compressed gas carbonate reservoirs necessitate the utilization of complex algorithms like RNN, GRU, and LSTM.
- Deep learning algorithms excel at autonomously extracting features from raw data, thereby enhancing our analysis of petrophysical data.
- Deep learning models demonstrate flexibility and adaptability, crucial for analyzing non-linear data distributions.

### Data collection

In the data collection phase of the research, the driven data are gathered from petrophysical assessments of the tight gas reservoir rock in the sedimentary basin of the Shulu Sag in the Bohai Bay Basin using various field measurement mechanisms like electrical conductivity, natural radiation, neutron radiation, and the propagation of sound waves in the rock environment. These measurements are commonly recorded as a part of engineering assessment programs of any oil and gas wells. the gathered dataset includes total of 33,950 data points, which arranged into 3397 rows of unique data. The parameters utilized in this research include the following inputs: Density index (RHOB), Corrected Gamma Ray (CGR), Sonic Transition Time (DT), Neutron porosity index (NPHI), Potassium content (POTA), Uranium content (URAN), Thorium content (THOR), and photoelectric coefficient index (PEF). The output parameter is Water Saturation (SW). Validity of SW data is confirmed through Schlumberger calculation control system. A summary of statistical data description in the research is presented in Table 1. This profiling gives a meaningful summary of the major characteristics of our dataset and provides a visual representation of the data's distribution.

**Table 1:** Data description for prediction of SW based on the proposed models.

Variables	DEPTH	RHOG	CGR	DT	NPHI	POTA	URAN	THOR	PEF
Mean	3984.75	2.76	36.37	67.20	15.06	1.35	1.95	4.63	4.49
Std. Deviation	149.38	0.08	29.54	9.69	6.79	1.10	1.28	3.91	1.43
Variance	22,308.39	0.01	872.16	93.96	46.04	1.20	1.63	15.27	2.06
Minimum	3601.12	2.71	1.06	51.12	0.02	0.01	0.02	0.13	1.91
Maximum	4368.37	2.96	121.40	117.20	46.67	5.27	10.18	16.52	11.82
Skewness	0.00	1.06	0.88	0.49	0.32	0.87	1.67	0.99	2.77
Kurtosis	− 1.20	− 0.41	− 0.41	− 0.03	0.19	− 0.27	4.80	− 0.19	13.05

### Shallow or classic machine learning models

This study aims to investigate the performance results of three shallow algorithm models with different mechanisms alongside deep learning models, with the goal of providing a comprehensive insight into benchmarking and comparing the performance of adapted deep machine algorithms with classic machine learning models:

Decision trees (DT) construct a tree-like model of decisions based on features, splitting data at each node to maximize information gain, making predictions by traversing the tree from root to leaf.

Support Vector Machines (SVM) classify data by finding the hyperplane that best separates classes, maximizing the margin between the nearest data points of different classes in a higher-dimensional space.

K-Nearest Neighbors (KNN) classify data based on the majority class of its k nearest neighbors in feature space, where distance metrics such as Euclidean or Manhattan distance are commonly used.

Each of these algorithms offers distinct approaches to classification, with decision trees focusing on hierarchical partitioning, SVM on finding optimal decision boundaries, and KNN on proximity-based classification.

### Deep neural networks architectures

The predictive core in solving the problem in this research is powered by three well-known deep learning algorithms include RNN, LSTM and GRU algorithms which They are networked with Adam's optimizer algorithm. The three proposed algorithms are all variants of neural network architectures used for sequence modeling and processing. While all three are based on the same fundamental concept of processing sequential data, they differ in their internal mechanisms and the way they handle long-term dependencies^2^. These models are actually an extension of each other, the most basic of which is RNN.

#### Recurrent neural networks (RNNs)

Recurrent Neural Networks (RNNs) are a specific category of artificial neural networks that is intended mainly for processing sequential data, such as speech, natural language, and time series. RNNs differ from ordinary feedforward neural networks as they possess a feedback mechanism, enabling them to capture temporal dependencies throughout the input data. This makes them particularly useful for tasks that involve predicting the next value in a sequence, such as speech recognition or language translation^46^.

The RNN algorithm operates by handling input features consecutively and utilizing a shared set of weights for every increment of time step. On each time recognized step increment, the network gets both an input vector and a hidden state vector, the latter of which includes information from the prior time increment^47^. The resulting vector is then passed through a nonlinear activation function, such as a sigmoid or hyperbolic tangent, to produce the current hidden state vector. The output of the RNN is typically produced by passing the final hidden state vector through a fully connected layer with a SoftMax activation function, which produces a probability distribution over the possible output values^48^. During training, the network's weights are adjusted using backpropagation through time, which involves propagating errors back through the sequence and updating the weights accordingly. The fundamentals, mathematical relations and the flow governing the RNN algorithm have been fully described by Tyagi et al^49^.

#### Long short-term memory (LSTM) algorithm

In 1997, LSTMs were introduced to tackle the problem of the disappearance of gradients in RNNs. Long Short-Term Memory, or LSTM for short, is a variety of deep learning neural network that is configured to simulate data sequence patterns, such as time series data or text-based natural language. At its core, an LSTM network consists of a series of interconnected nodes, known as neurons, arranged in a specific way. Every individual neuron within the neural network assumes the responsibility of analyzing a particular information component obtained from the preceding layer and subsequently relaying it to the succeeding layer in the network^50^. This information is transformed by a set of learnable weights and biases before being fed into the next neuron in the next layer. What makes an LSTM unique is the architecture of each neuron within the network, which includes multiple "gates" that determine what information is kept and what is discarded. The ensemble of gates comprises the input gate, forget gate, and output gate, where each of these gates assumes a particular role in regulating the passage of information across the network^51^. The input gate determines how much new information from the current input should be allowed to pass through to the next layer. In contrast, the forget gate regulates the extent to which information from prior inputs is deemed unnecessary and therefore disregarded or eliminated. Finally, the output gate decides what part of the current information should be sent to the next layer in the network. LSTM exhibits superiority over alternative neural networks because it allows for modeling more complex sequence data with much better accuracy compared to others. It is capable of processing longer sequences of data, preventing the vanishing gradient problem that occurs with traditional neural networks. LSTM networks possess the capability to acquire long-term dependencies within data with considerable ease. As a consequence of this ability, they prove to be advantageous for various tasks such as sentiment analysis, machine translation, speech and handwriting recognition. The fundamentals, mathematical relations and the flow governing the LSTM algorithm have been fully described by Sherstinsky et al^52^.

#### Gated recurrent unit (GRU) algorithm

The Gated Recurrent Unit (GRU) algorithm is a special form of RNN that is used to process sequential data. It was first introduced by Cho et al. in 2014 as an improvement over the standard RNN architecture. GRUs were introduced as a simpler alternative to LSTMs. They have a similar structure to LSTMs, but with fewer gates^53^. The GRU architecture has gating mechanisms that allow it to selectively remember or forget information from the previous time step Chung et al^54^. The GRU architecture is composed of three distinct gates, namely, the input gate, reset gate, and update gate. The input gate regulates the quantity of fresh data that will be incorporated into the memory cell, the reset gate identifies which elements of the state vector must be discarded, whereas the update gate governs the influence of the prior state and the current input on the output. The activation function used in GRUs is typically a hyperbolic tangent function or a rectified linear unit (ReLU). The GRU works by processing input data sequences one element at a time, while maintaining an internal state that stores information from previous elements^55^. Compared to the standard RNN model, GRUs have been shown to have better performance when dealing with long-term dependencies in sequential data. This is due to the gating mechanism that allows them to selectively decide what information is important to keep and what can be safely forgotten. Additionally, they have fewer parameters to train than the LSTM model, which leads to faster training times. They also can be used for both sequence-to-sequence learning and sequence classification tasks. The fundamentals, mathematical relations and the flow governing the GRU algorithm have been fully described by Sachin et al^56^.

#### Adam optimizer algorithm

The Adam optimizer algorithm is a widely used optimization algorithm for stochastic gradient descent (SGD), which is used to update the weight parameters in DL models. It was first proposed by Kingma and Ba^57^.

The Adam optimizer operates by estimating the first and second moments of the gradient. The initial moment estimate is applied to compute the gradient's mean, while the second moment estimate calculates its variance. The algorithm combines these two estimates to create a new adaptive learning rate that takes into account both the gradient and the variance. This helps the optimizer to converge faster and also avoid getting stuck in local minima or saddle points^58^. The Adam optimizer has several advantages over other optimization algorithms such as SGD and other variants, including: Adam uses an adaptive learning rate, which means it automatically adjusts the learning rate of each parameter based on its importance in the model. This ensures that parameters with a large gradient will have a smaller learning rate, while those with a small gradient will have a larger learning rate. Adam also incorporates the concept of momentum, which helps to smooth the updates made to the model. This prevents oscillations and overshooting of the minimum, ensuring that the model converges as soon as possible. As mentioned earlier, the Adam optimizer converges faster than other optimization algorithms. This is because it uses a combination of both the first and second moments, allowing it to adapt to the contour of the optimization surface. Adam optimizers are also computationally efficient, making them ideal for use in large-scale DL models.

#### K-fold cross validation technique

K-fold cross-validation is a technique used in machine learning to assess the performance of a model^59^. The method involves partitioning a given dataset into K folds or segments and subsequently fitting the model K times. During each of these iterations, a distinct fold is reserved as the validation set, while the other folds serve as training data. This repetition continues until each of the K segments has been utilized as the testing set once. The resulting accuracy metric is then determined by averaging the accuracy results from all iterations. K-Fold cross-validation ensures that every part of the dataset is used as both training and testing data^60,61^. The main goal of K-Fold cross-validation is to provide a more accurate estimate of the model's performance by reducing bias and variance^62^. By splitting the data into multiple folds and training the model on different subsets of the data, K-Fold cross-validation helps in reducing the dependency of the model on a particular training set^63^. This helps in creating a more robust and generalizable model that can perform well on unseen data. By testing the model on multiple subsets of the data, K-Fold cross-validation helps in capturing the variability in the data and provides a more accurate assessment of the model’s performance^64^. This can help in identifying potential issues with the model and improving its overall accuracy. One of the key advantages of K-Fold cross-validation is its ability to prevent overfitting. Overfitting occurs when a model is too complex and captures noise in the training data, leading to poor performance on unseen data. By training the model on multiple subsets of the data and averaging the results, K-Fold cross-validation helps in creating a more generalized model that is less likely to overfit the training data.

**Figure 3:**
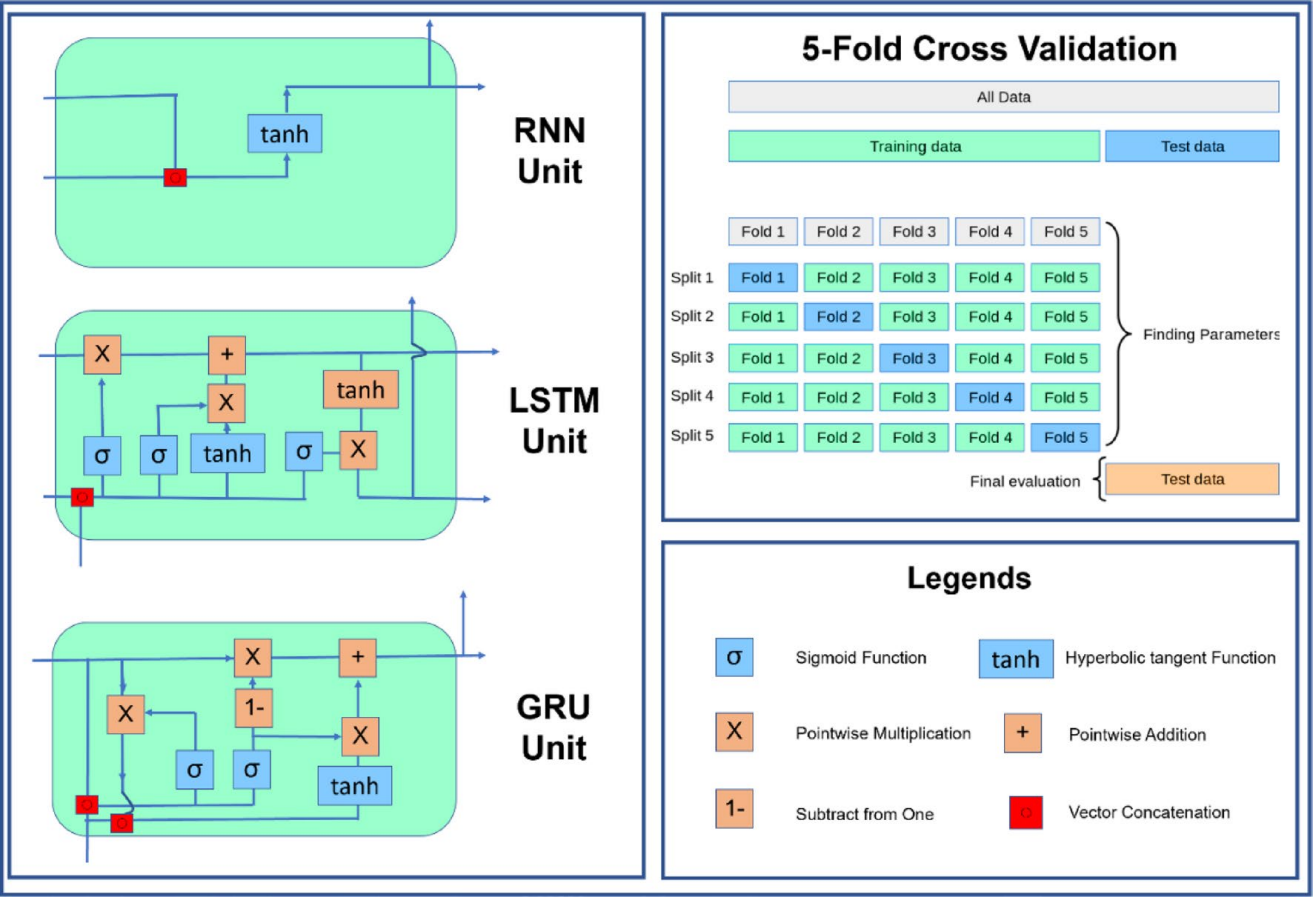
The structure of RNN, LSTM, and GRU units and cross-validation technique to accredit the proposed models.

Figure 3 schematically shows the process of implementing the K-Fold cross-validation technique and the participation of all the divided 5-parts (K = 5) in the model training process. Figure 3 also shows the structure of RNN, LSTM, and GRU units.

### Model implementation and reproducibility

**Figure 4:**
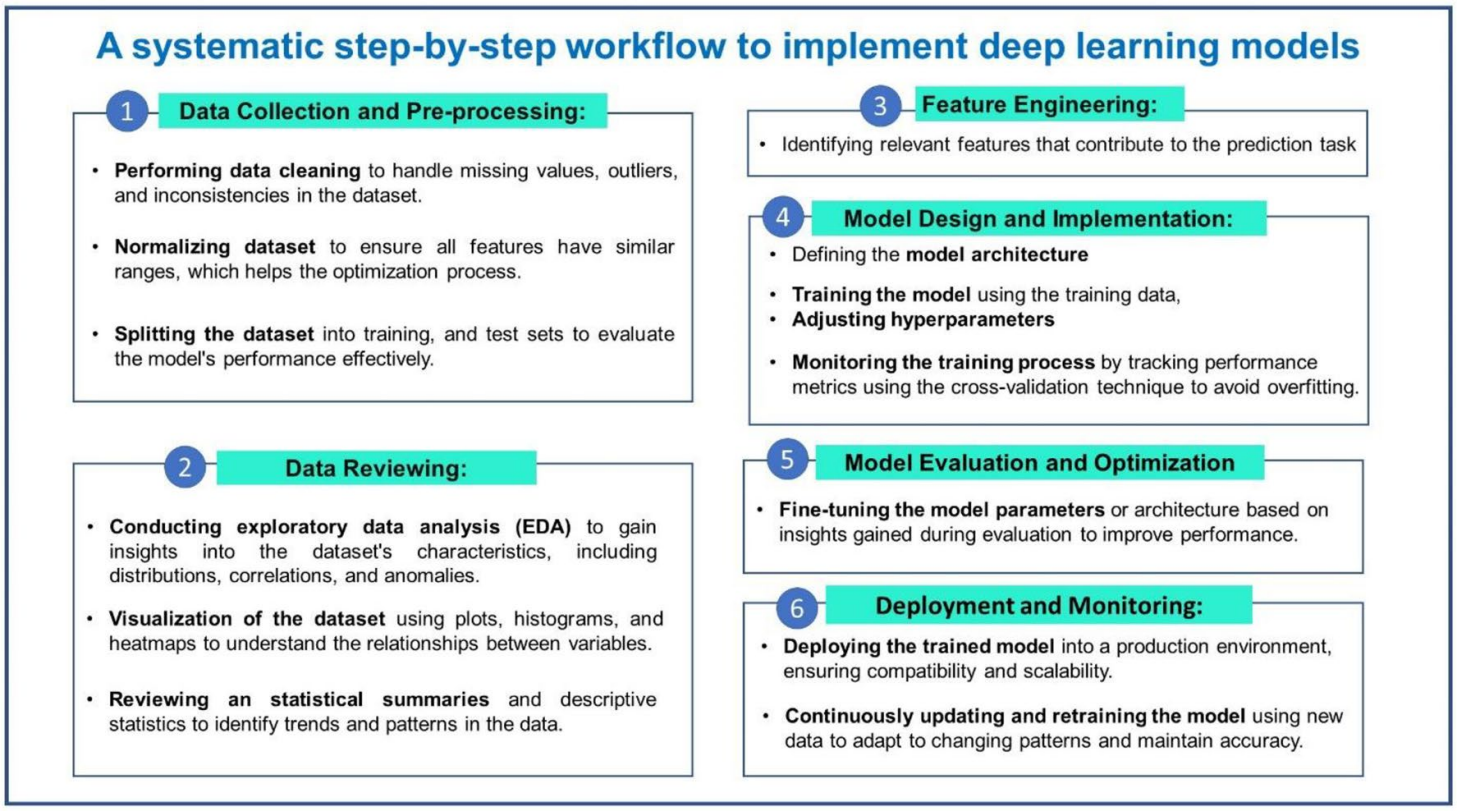
Systematic steps workflow to implement deep learning models.

To assess the efficacy of three proposed models within a similar algorithmic framework, all three optimized deep learning algorithm models were adapted to Adam's optimizer for a numerical sequencial series data set comprising 12 inputs and 1 output. The proposed architecture for the models comprises an input layer containing 12 input nodes, each representing an input feature in the time series. The hidden layer comprises two layers of RNN, LSTM, and GRU units in matching algorithms. The number of hidden layers may be adjusted based on the complexity of the problem, but two or three layers are typically effective. Two hiden layer was selected based on experience and problem complexity, with 64 units and a ReLU activation function included. Finally, the output layer comprises a node for predicted output, which may utilize a linear activation function for regression problems since it is a numerical time series data set. The second hidden layer is followed by a dropout layer to regularize the model. Finally, a dense output layer with a sigmoid activation function is added and dropout regularization is considered for model overfitting concerns. the models compiled using the adam optimizer and binary-crossentropy loss function. The models also supported by fivefold cross-validation function, and use a batch size of 32 and train for 100 epochs in training phase. Figure 4 shows the systematic steps workflow to implement deep learning models.

## Result and discussion

The aim of this research is to develop precise and dependable machine learning models for the prediction of SW (Water Saturation) using three DL and three SL techniques: LSTM, GRU, RNN, SVM, KNN and DT. These models were trained on an extensive dataset comprising various types of log data. The findings of our investigation illustrate the efficacy of data-driven machine learning models in SW prediction, underscoring their potential for a wide range of practical applications.

When evaluating and comparing algorithms, researchers must take into account several crucial factors. Accuracy and disparities in prediction are among the most significant considerations. To evaluate these factors, researchers can utilize various criteria, including Eqs. 1–6. The Mean Percentage Error (MPE) calculates the average difference between predicted and actual values as a percentage, while the Absolute Mean Percentage Error (AMPE) measures the absolute difference between them. Additionally, the Standard Deviation (SD) determines the variability of data points around the mean. Moreover, the Mean Squared Error (MSE) and Root Mean Squared Error (RMSE) quantify the mean and root mean squared differences between predicted and actual values, respectively. Lastly, the R^2^ metric assesses the fraction of diversity in the reliant variable that can be accounted for by the autonomous variable.

1 $$\text{MPE}=\frac{{{\sum }_{\text{i}=1}^{\text{n}}(\frac{{SWE}_{(\text{Meas}.)}-{SWE}_{(\text{Pred}.)}}{{SWE}_{(\text{Meas}.)}}\text{x }100)}_{\text{i}}}{\text{n}}$$

2 $$\text{AMPE}=\frac{{\sum }_{\text{i}=1}^{\text{n}}\left|{(\frac{{SWE}_{(\text{Meas}.)}-{SWE}_{(\text{Pred}.)}}{{SWE}_{(\text{Meas}.)}}\text{x }100)}_{\text{i}}\right|}{\text{n}}$$

3 $$\text{SD}=\sqrt{\frac{{\sum }_{\text{i}=1}^{\text{n}}{({\left(\frac{1}{\text{n}}\sum_{\text{i}=1}^{\text{n}}\left({{SWE}_{\text{Meas}.}}_{\text{i}}-{{SWE}_{\text{Pred}.}}_{\text{i}}\right)\right)}_{\text{i}}-(\frac{1}{\text{n}}\sum_{\text{i}=1}^{\text{n}}\left({{SWE}_{\text{Meas}.}}_{\text{i}}-{{SWE}_{\text{Pred}.}}_{\text{i}}\right))\text{imean})}^{2}}{\text{n}-1}}$$

4 $$\text{MSE}=\frac{1}{\text{n}}\sum_{\text{i}=1}^{\text{n}}{\left({{SWE}_{\text{Meas}.}}_{\text{i}}-{{SWE}_{\text{Pred}.}}_{\text{i}}\right)}^{2}$$

5 $$\text{RMSE}=\sqrt{\frac{1}{\text{n}}\sum_{\text{i}=1}^{\text{n}}{\left({{SWE}_{\text{Meas}.}}_{\text{i}}-{{SWE}_{\text{Pred}.}}_{\text{i}}\right)}^{2}}$$

6 $${\text{R}}^{2}=1-\frac{\sum_{\text{i}=1}^{\text{N}}{({{SWE}_{\text{Pred}.}}_{\text{i}}-{{SWE}_{\text{Meas}.}}_{\text{i}})}^{2}}{\sum_{\text{i}=1}^{\text{N}}{({{SWE}_{\text{Pred}.}}_{\text{i}}-\frac{{\sum }_{\text{I}=1}^{\text{n}}{{SWE}_{\text{Meas}.}}_{\text{i}}}{\text{n}})}^{2}}$$

In order to forecast SW, three DL and three SL techniques: LSTM, GRU, RNN, SVM, KNN and DT, were used in this study. Each algorithm underwent individual training and testing processes, followed by independent experiments. To ensure the accuracy of the predictions, the dataset was carefully divided into three subsets. The training subset accounted for 70% of the data records, while 30% was allocated for independent testing.

Choosing the most suitable algorithm for a specific task is a crucial undertaking within the realm of data analysis and machine learning. Therefore, this research aimed to assess and compare the performance of multiple LSTM, GRU, and RNN algorithms in predicting SW. The outcomes of these algorithms, utilizing the train data values, as well as the test, have been meticulously documented and presented in Table 2. By analyzing the results, researchers can gain insights into the effectiveness of each algorithm and make informed decisions about their implementation in practical applications.

**Table 2:** Determination of error parameters for prediction of SW based on three DL and three SL techniques: LSTM, GRU, RNN, SVM, KNN and DT.

Dataset	Models	MPE	AMPE	MSE	RMSE	R^2^
	Units	(%)	(%)	(-)	(-)	(-)
Train Data	GRU	0.0770	1.5217	0.0001	0.0115	0.9980
	LSTM	− 0.0237	2.4073	0.0003	0.0178	0.9823
	RNN	0.0942	2.6021	0.0004	0.0208	0.9771
	SVM	0.1478	5.1368	0.0025	0.0499	0.8375
	KNN	− 0.0987	5.7365	0.0028	0.0534	0.8109
	DT	0.1789	6.8280	0.0037	0.0609	0.7917
Test Data	GRU	− 0.1492	2.0320	0.0004	0.0198	0.9973
	LSTM	− 0.1388	3.1136	0.0008	0.0284	0.9725
	RNN	− 0.0201	4.0613	0.0016	0.0399	0.9701
	SVM	− 0.1664	6.1642	0.0036	0.0599	0.8050
	KNN	0.0997	7.4575	0.0048	0.0694	0.7873
	DT	− 0.1758	8.1936	0.0053	0.0730	0.7289

The results from the test data are presented in Table 2, highlighting the excellent performance of the RMSE, MPE and AMPE metrics for the GRU algorithm, with values of 0.0198, − 0.1492 and 2.0320, respectively. Similarly, for the LSTM algorithm, the corresponding values are 0.0284, − 0.1388 and 3.1136, while for the RNN algorithm, they are 0.0399, − 0.0201 and 4.0613, respectively. For SVM, KNN and DT these metrics are includes: 0.0599, − 0.1664 and 6.1642; 0.7873, 0.0997 and 7.4575; 0.7289, − 0.1758 and 8.1936. The results show the GRU model has high accuracy than other algorithms.

**Figure 5:**
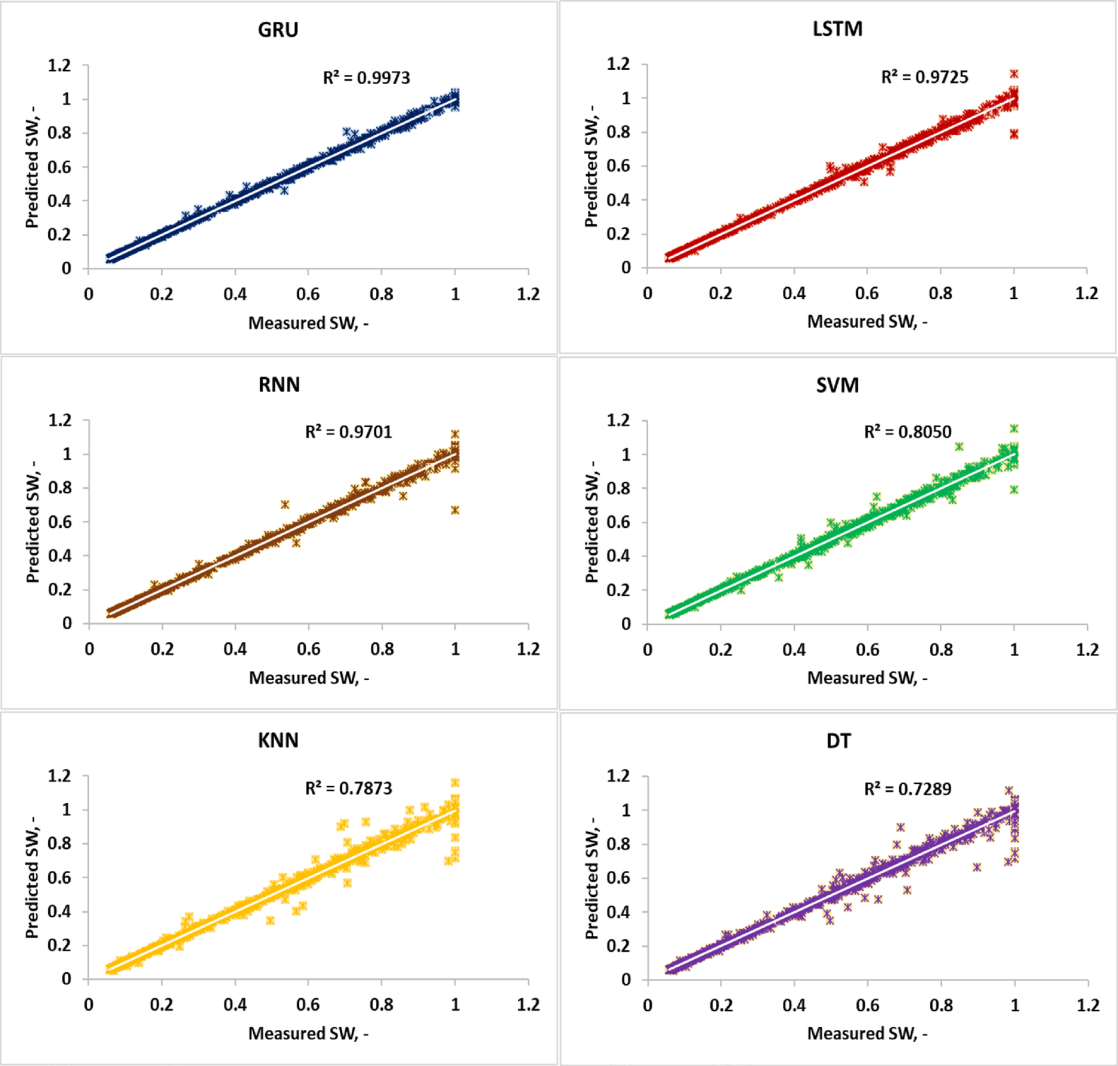
Cross plot for predicting SW using three DL algorithms such as RGU, LSTM and RNN for test data.

The R^2^ parameter is a crucial statistical measure for evaluating and comparing different models. It assesses the adequacy of a model by quantifying the amount of variation in the outcome variable that can be clarified by the explanatory variables. In this study, Fig. 5 illustrates cross plots for predicting SW values based on the train and test data, demonstrating significantly higher prediction accuracy compared to the other evaluated models. Additionally, Fig. 5 confirms that the RGU model exhibits superior prediction accuracy compared to the LSTM and RNN models.

To assess the precision of the GRU model, the results presented in Table 2 and Fig. 5 were carefully analyzed for the train and test data. The analysis revealed that the GRU algorithm achieved low errors for SW, with RMSE values of 0.0198 and R-square values of 0.9973. The R^2^ values provided serve as quantitative metrics assessing the predictive prowess of ML models. The R^2^, denoting the coefficient of determination, gauges the extent to which the variance in the dependent variable can be foreseen from the independent variable(s), essentially showcasing how well the model aligns with observed data points. The R^2^ values for the GRU, LSTM, RNN, SVM, KNN and DT models stand at 0.9973, 0.9725, 0.9701, 0.8050, 0.7873 and 0.7289 respectively, reflecting their respective accuracy and reliability in predicting SW levels. Figure 5 shows the cross plot for predicting SW using three DL algorithms such as RGU, LSTM, and RNN for test data. The GRU model's notably high R^2^ of 0.9973 underscores its exceptional correlation between predicted and observed SW values, implying that nearly 99.73% of SW data variance can be elucidated by its predictions, showcasing its precision and reliability in SW prediction tasks. Comparatively, the LSTM and RNN models, with R^2^ values of 0.9725 and 0.9701 respectively, also exhibit strong predictive capabilities, albeit slightly lower than the GRU model. These findings underscore the GRU model's superiority in SW prediction, attributed to its adeptness in capturing intricate temporal dependencies within SW data, thereby yielding more accurate predictions.

**Figure 6:**
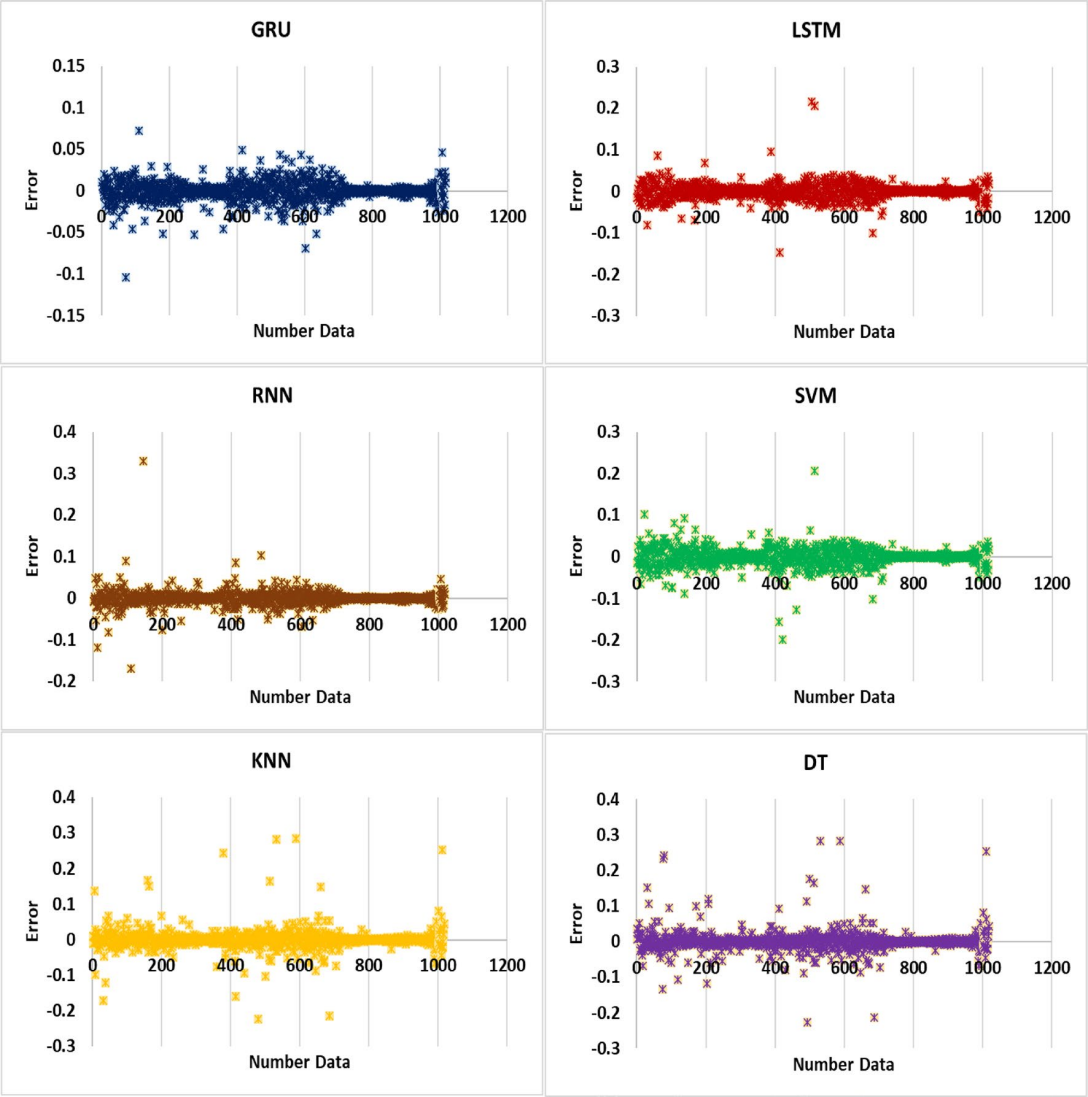
Error points for predicting SW using three DL and three SL algorithms such as GRU, LSTM, RNN, SVM, KNN and DT for test data.

Figure 6 provides a visual representation of the calculation error for the test data, illustrating the error distribution for predicting SW using three DL algorithms (GRU, LSTM, and RNN). The plotted coordinates in the figure depict the error range for each algorithm. For the GRU algorithm, the error range is observed to be between − 0.0103 and 0.0727. This indicates that the predictions made by the GRU model for the test data exhibit a relatively small deviation from the actual SW values within this range. In contrast, the LSTM algorithm demonstrates a slightly wider error range, ranging from − 0.146 to 0.215. This suggests that the predictions generated by the LSTM model for the test data exhibit a somewhat higher variability and may deviate from the actual SW values within this broader range.

Similarly, the RNN algorithm exhibits an error range between − 0.222 and 0.283. This indicates that the predictions made by the RNN model for the test data show a larger spread and have the potential to deviate more significantly from the actual SW values within this range. By visually comparing the error ranges for the three DL algorithms, it becomes apparent that the GRU algorithm achieves a narrower range and thus demonstrates better precision and accuracy in predicting SW for the test data. Conversely, the LSTM and RNN algorithms exhibit broader error ranges, indicating a higher degree of variability in their predictions for the same dataset. These findings further support the conclusion that the GRU algorithm outperforms the LSTM and RNN algorithms in terms of SW prediction accuracy, as it consistently produces predictions with smaller errors and tighter error bounds.

**Figure 7:**
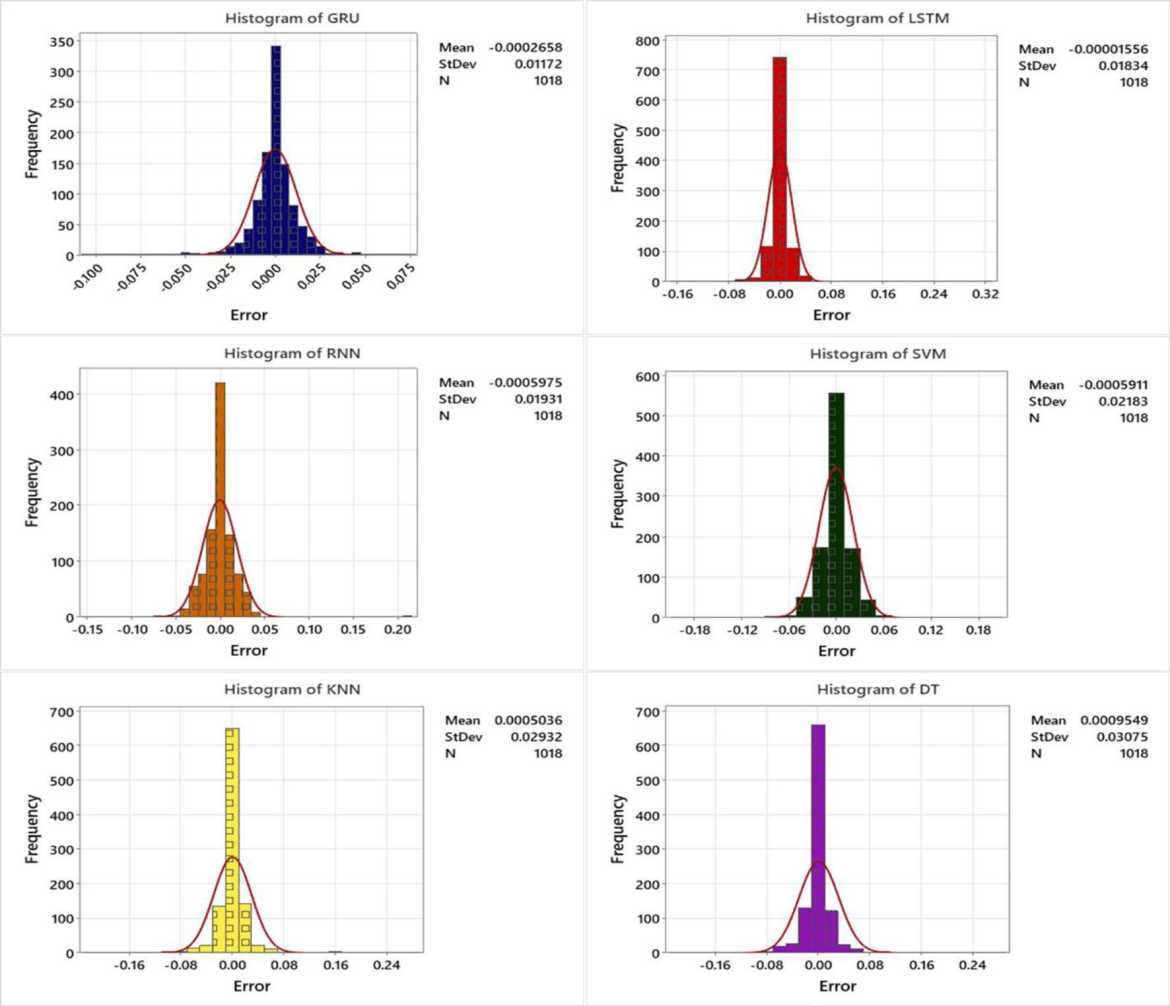
Histogram plot for SW prediction using three DL and three SL algorithms such as GRU, LSTM, RNN, SVM, KNN and DT.

Figure 7 presents an error histogram plot, depicting the prediction errors for SW using three DL and three SL algorithms such as GRU, LSTM, RNN, SVM, KNN and DT. Each histogram represents the distribution of prediction errors for each algorithm, displaying a normal distribution centered around zero with a relatively narrow spread and no noticeable positive or negative bias. This plot enables a comprehensive analysis of the algorithms' performance and aids in determining the best algorithm with a normal error distribution. Upon careful investigation, it becomes evident that the GRU algorithm exhibits a superior normal distribution of data compared to the other algorithms. The GRU algorithm's performance is characterized by a more accurate standard deviation and a narrower spread of prediction errors. This indicates that the GRU algorithm consistently produces more precise and reliable predictions for SW. By comparing the results presented in Table 2 and analyzing the error histogram plot in Fig. 7, we can conclude that the performance accuracy of the algorithms can be ranked as follows: GRU > LSTM > RNN > SVM > KNN > DT.

**Figure 8:**
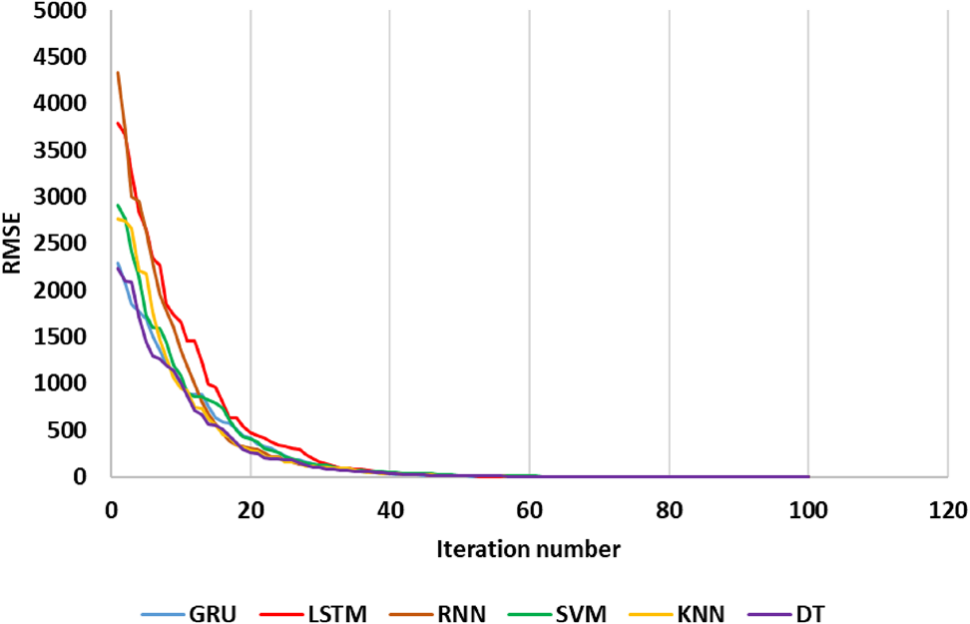
Iteration plot for SW prediction using three DL and three SL algorithms such as GRU, LSTM, RNN, SVM, KNN and DT.

Figure 8 illustrates the error rate of the three DL and three SL algorithms such as GRU, LSTM, RNN, SVM, KNN and DT as a function of iteration for SW prediction. The findings of this study indicate that the GRU and LSTM algorithms initially exhibit higher error values that progressively decrease over time. However, this pattern is not observed in the RNN algorithm. Upon analyzing the figure, it becomes evident that the LSTM algorithm achieves higher accuracy than the other algorithms at the beginning of the iteration. At the threshold of 10 iterations, the LSTM algorithm surpasses the GRU algorithm with a lower error value. However, in the subsequent iterations, specifically at iteration 31, the GRU algorithm outperforms the LSTM algorithm with superior performance accuracy. In contrast, the RNN algorithm shows a consistent decrease in performance accuracy from the start to the end of the iterations, without displaying significant fluctuations. When focusing on the zoomed-in portion of the figure, specifically repetitions 85–100, the ongoing performance trends of these algorithms become more apparent. It is evident from the analysis that the GRU algorithm consistently outperforms the other algorithms in terms of performance accuracy. The LSTM algorithm follows, with a decrease in accuracy over the iterations. On the other hand, the RNN algorithm exhibits a declining performance accuracy without any notable changes or fluctuations. These findings emphasize the superiority of the GRU algorithm in terms of performance accuracy when compared to the LSTM and RNN algorithms. The GRU algorithm consistently maintains a higher level of accuracy throughout the iterations, while the LSTM and RNN algorithms experience fluctuations and decreasing accuracy over time.

Pearson's coefficient (R) is a widely used method for assessing the relative importance of input-independent variables compared to output-dependent variables, such as SWL. The coefficient ranges between − 1 and + 1 and represents the strength and direction of the correlation. A value of + 1 indicates a strong positive correlation, -1 indicates a strong negative correlation, and a value close to 0 indicates no correlation. Equation 7 illustrates the calculation of Pearson's correlation coefficient, which is a statistical measure of the linear relationship between two variables. It allows researchers to quantify the extent to which changes in one variable are associated with changes in another variable. By applying Pearson's coefficient, researchers can determine the level of influence that input-independent variables have on the output-dependent variable, SWL.

7 $$R=\frac{\sum_{i=1}^{n}({Z}_{i}-\overline{Z })({Q}_{i}-\overline{Q })}{\sqrt{{\sum }_{i=1}^{n}{({Z}_{i}-\overline{Z })}^{2}}\sqrt{{\sum }_{i=1}^{n}{({Q}_{i}-\overline{Q })}^{2}}}$$

A coefficient of + 1 indicates a perfect positive correlation, suggesting that the input-independent variables have the greatest positive impact on the output-dependent variable. Conversely, a coefficient of − 1 represents a perfect negative correlation, indicating that the input-independent variables have the greatest absolute impact on the output-dependent variable. When the coefficient is close to 0, it suggests that there is no significant correlation between the variables, indicating that changes in the input-independent variables do not have a substantial effect on the output-dependent variable. Pearson's correlation coefficient is a valuable tool for assessing the relationship between variables and understanding their impact. It provides researchers with a quantitative measure to determine the relative importance of input-independent variables compared to the output-dependent variable, SWL.

**Figure 9:**
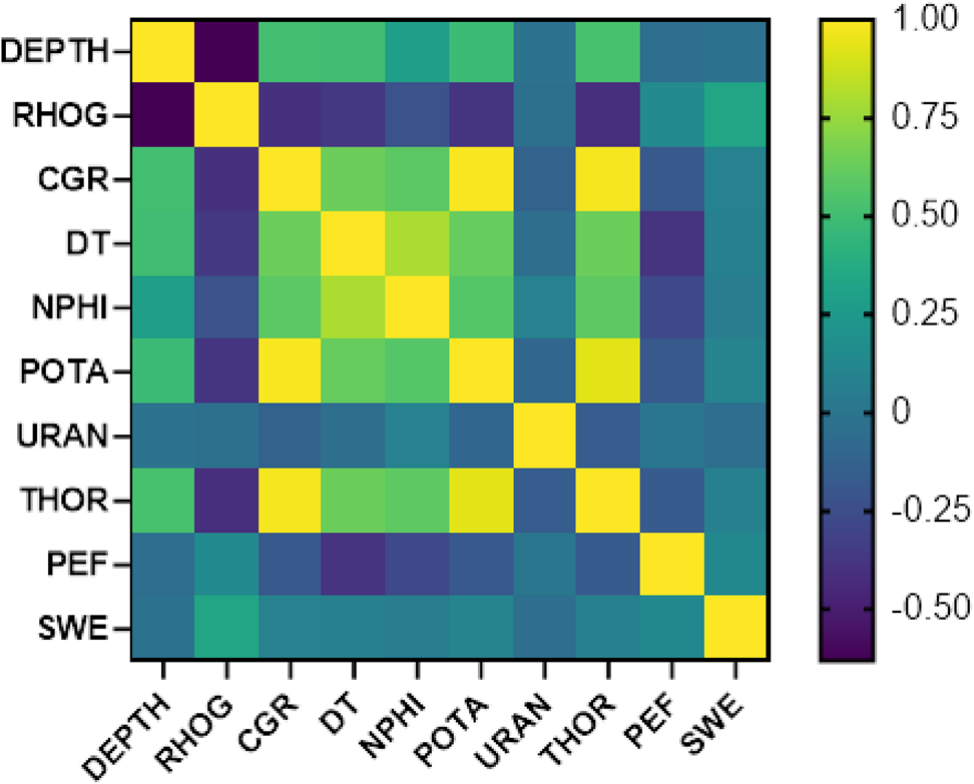
Heat map plot for SW prediction using three DL and three SL algorithms such as GRU, LSTM, RNN, SVM, KNN and DT.

By heat map shows Fig. 9, a comparison of Pearson correlation coefficients can be made to gain insights into the relationship between input variables and SW. The results reveal several significant correlations between the variables. Negative correlations are observed with URAN and DEPTH, indicating an inverse relationship with SW. This suggests that higher values of URAN and DEPTH are associated with lower SW values. On the other hand, positive correlations are observed with CGR, DT, NPHI, POTA, THOR, and PEF. These variables show a direct relationship with SW, meaning that higher values of CGR, DT, NPHI, POTA, THOR, and PEF are associated with higher SW values. The comparison of Pearson correlation coefficients provides valuable insights into the relationship between input variables and SW.

These findings can be utilized to develop predictive models of SW based on the input variables. By incorporating the correlations into the models, researchers can enhance their accuracy and reliability in predicting SW values. The expression of the relationships between the input variables and SW in the form of Eq. 8 allows for quantitative analysis of the data. This equation provides a mathematical representation of the correlations, enabling researchers to quantitatively evaluate the impact of the input variables on SW.

8 $$SWE=\propto \left(\text{CGR},\text{ DT},\text{ NPHI},\text{ POTA},\text{ THOR},\text{ PEF}\right) and SWE=\propto \frac{1}{\left(URAN, DEPTH\right)}$$

## Conclusion

This study addresses the fundamental need for accurate prediction of water saturation levels in tight gas carbonate reservoirs using specialized deep learning algorithms tailored to sequential pattern recognition. Such prediction is crucial for optimizing hydrocarbon production and mitigating challenges associated with reduced permeability and pore throat blockages. In contrast to traditional machine learning models that use statistical approximation techniques and gradient optimization methods, this study leverages structured and adapted deep learning algorithms which are specifically designed to handle sequential and temporal patterns, making them ideal for analyzing time series geological data and repetitive time-dependent sediment sequences. A comprehensive dataset consisting of 3,397 unique data entries was utilized in this study. The input parameters included Density index (NPHI), Compression Gamma Ray (CGR), Sonic Transition Time (DT), Neutron index (NPHI), Potassium (POTA), Uranium (URAN), Thorium (THOR), and photoelectric coefficient index (PEF). The output parameter of interest was Water Saturation (SW). Through extensive analysis, it was discovered that deep/shallow machine learning (DL) algorithms, namely Gated Recurrent Unit (GRU), Recurrent Neural Networks (RNN), Long Short-Term Memory (LSTM), Support Vector Machine (SVM), K-nearest neighbor (KNN) and Decision tree (DT) can be effectively used to accurately predict SW. Additionally, Pearson correlation coefficient analyses revealed that certain input variables, such as THOR and PEF, exhibited a negative and indirect relationship with SW, while CGR, DT, NPHI, POTA, THOR, and PEF displayed positive correlations. The GRU model, applied to the entire dataset, achieved impressive SW prediction accuracy, as evidenced by an RMSE of 0.0198 and an R^2^ value of 0.9973. The GRU algorithm, a powerful and reliable machine learning tool, excels in processing data points and is particularly adept at learning and identifying patterns in large datasets. The RGU algorithm offers several advantages over LSTM, RNN, SVM, KNN and DT algorithms for predicting SW. It achieves higher accuracy with lower error values and improved performance metrics. The GRU algorithm effectively handles long-term dependencies, making it suitable for scenarios where such dependencies are significant. It also provides faster training and inference, reduces overfitting, has a simpler architecture for better interpretability, and utilizes memory efficiently. These advantages make the GRU algorithm a preferred choice for accurate and reliable SW predictions, especially in applications that require quick response times and limited computational resources.

## Data Availability

Correspondence and requests for materials should be addressed to J.X.

## Abbreviations

AI: Artificial intelligence
AMPE: Absolute mean percentage error
ANFIS: Adaptive neuro-fuzzy inference system
ANN: Artificial neural networks
BMA: Bayesian model averaging
CGR: Corrected gamma ray
DL: Deep learning
DT: Sonic transition time
DT: Decision tree
FNO: Fourier neural operator
GBM: Generalized boosted regression models
GMDH: Group method of data handling
GPR: Gaussian process regression
GRU: Gated recurrent unit
LASSO: Least absolute shrinkage and selection operator regression
LS-SVM: Least-squares support vector machine
ML: Machine learning
MLP: Multilayer perceptron
MPE: The mean percentage error
MRGC: Multi-dimensional dot-pattern recognition
MSE: Mean squared error
NPHI: Neutron porosity index
PDE: Partial differential equations
PEF: Photoelectric coefficient index
PNN: Probabilistic neural networks
POTA: Potassium content
PR: Polynomial regression
R: Pearson's coefficient
R^2^: R-squared
ReLU: Rectified linear unit
RF: Random forest
RHOB: Density index
RMSE: Root mean squared error
RNN: Recurrent neural networks
SD: Standard deviation
SGAM: Smooth generalized additive models
SGD: Stochastic gradient descent
SL: Shallo learning
SOM: Self-organizing map
SVM: Support vector machine
SW: Water saturation
THOR: Thorium content
URAN: Uranium content
XGBoost: Extreme gradient boosting regression
